# Crystal structure of (*E*)-*N*-cyclo­hexyl-2-(2-hy­droxy-3-methyl­benzyl­idene)hydrazine-1-carbo­thio­amide

**DOI:** 10.1107/S2056989019008946

**Published:** 2019-06-28

**Authors:** Md. Azharul Arafath, Huey Chong Kwong, Farook Adam

**Affiliations:** a Department of Chemistry, Shahjalal University of Science and Technology, Sylhet 3114, Bangladesh; b School of Chemical Sciences, Universiti Sains Malaysia, Penang 11800 USM, Malaysia

**Keywords:** crystal structure, hydrazinecarbo­thio­amide, Schiff base, inter­molecular inter­actions

## Abstract

The asymmetric unit of the title compound, C_15_H_21_N_3_OS, comprises of two crystallographically independent mol­ecules (*A* and *B*). Each mol­ecule consists of a cyclo­hexane ring and a 2-hy­droxy-3-methyl­benzyl­idene ring bridged by a hydrazinecarbo­thio­amine unit. Both mol­ecules exhibit an *E* configuration with respect to the azomethine C=N bond. There is an intra­molecular O—H⋯N hydrogen bond in each mol­ecule forming an *S*(6) ring motif. The cyclo­hexane ring in each mol­ecule has a chair conformation. The benzene ring is inclined to the mean plane of the cyclo­hexane ring by 47.75 (9)° in mol­ecule *A* and 66.99 (9)° in mol­ecule *B*. The mean plane of the cyclo­hexane ring is inclined to the mean plane of the thio­urea moiety [N—C(=S)—N] by 55.69 (9) and 58.50 (8)° in mol­ecules *A* and *B*, respectively. In the crystal, the *A* and *B* mol­ecules are linked by N—H⋯S hydrogen bonds, forming ‘dimers’. The *A* mol­ecules are further linked by a C—H⋯π inter­action, hence linking the *A*–*B* units to form ribbons propagating along the *b*-axis direction. The conformation of a number of related cyclo­hexa­nehydrazinecarbo­thio­amides are compared to that of the title compound.

## Abstract 2

The title Schiff base compound, consisting of a cyclo­hexane and a 2-hy­droxy-3-methyl­benzyl­idene ring bridged by a hydrazinecarbo­thio­amine moiety, crystallizes with two independent mol­ecules in the asymmetric unit. In the crystal, the mol­ecules are linked by N—H⋯S hydrogen bonds and C—H⋯π inter­actions, forming ribbons along the [010] direction.

## Chemical context   

Schiff bases are significant agents in both organic and inorganic chemistry, and are widely used in biological applications, particularly for anti­cancer screening (Ziessel, 2001 ▸; Salam *et al.*, 2012*a* ▸; Arafath *et al.*, 2017*b* ▸). They have attracted a great deal of attention because of the presence of hard and soft atoms together in one mol­ecule. Thio­semicarbazone Schiff base compounds have soft sulfur and hard nitro­gen as well hard oxygen atoms (Mohamed *et al.*, 2009 ▸). These Schiff base compounds are of special inter­est because of their diversity in coordinating to hard and soft metals using the hard and soft coordinating sites such as NSO (Arion *et al.*, 2001 ▸; Leovac & Češljević, 2002 ▸; Chandra & Sangeetika, 2004 ▸; Singh *et al.*, 2000 ▸; Gerbeleu *et al.*, 2008 ▸; Mohamed *et al.*, 2009 ▸). Many Schiff base compounds and their complexes with transition metals have wide biological and pharmaceutical applications (Padhyé & Kauffman, 1985 ▸; Salam *et al.*, 2012*b* ▸). Thio­semicarbazones having ONS-coordinating sites are important for coordination chemistry because of their strong bonding ability with transition metals (Rayati *et al.*, 2007 ▸; Alomar *et al.*, 2009 ▸; Vieites *et al.*, 2009 ▸; Siddiki *et al.*, 2012 ▸).

## Structural commentary   

**Figure 1:**
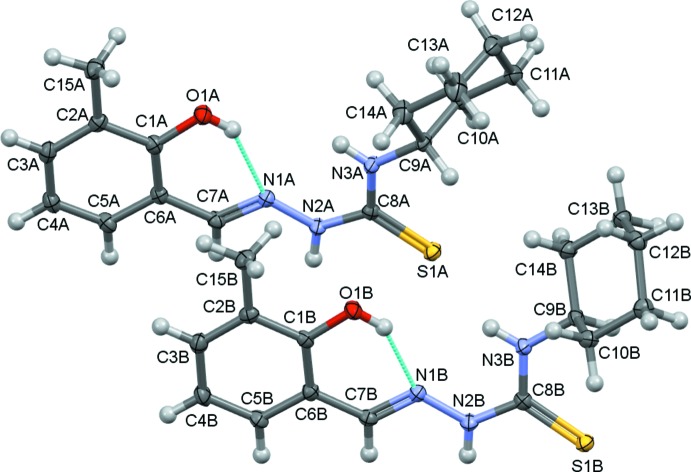
A view of the mol­ecular structure of the two independent mol­ecules (*A* and *B*) of the title compound, with the atom labelling. Displacement ellipsoids are drawn at the 50% probability level. The intra­molecular O—H⋯N hydrogen bonds (Table 1 ▸) are shown as dashed cyan lines.

**Figure 2:**
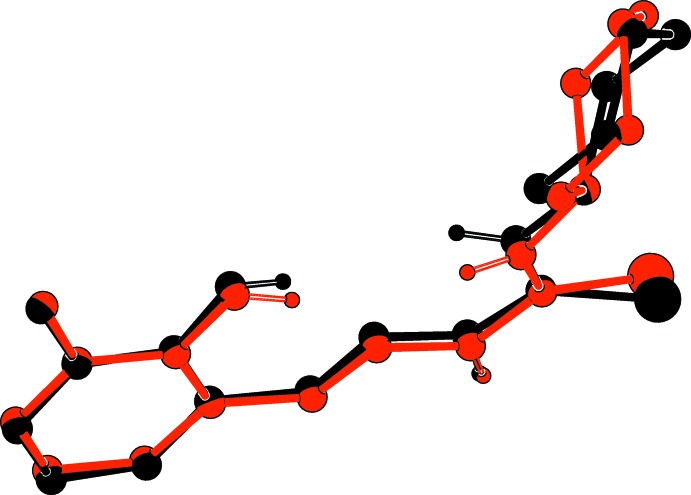
An AutoMolFit figure (*PLATON*; Spek, 2009 ▸) of inverted mol­ecule *B* (red) on mol­ecule *A* (black).

The asymmetric unit of the title compound consists of two crystallographic independent mol­ecules (*A* and *B*), as illustrated in Fig. 1 ▸. In each mol­ecule a cyclo­hexane ring and a 2-hy­droxy-3-methyl­benzyl­idene ring are inter­connected by a hydrazinecarbo­thio­amine bridge. Both mol­ecules exhibit an *E* configuration with respect to the azomethine C7=N1 bond, and in each mol­ecule there is an intra­molecular O—H⋯N hydrogen bond forming an *S*(6) ring motif (Table 1 ▸and Fig. 1 ▸). The best AutoMolFit (*PLATON*; Spek, 2009 ▸) image of the two mol­ecules, *viz*. inverted mol­ecule *B* (red) on mol­ecule *A* (black), which has an r.m.s. deviation of 0.654 Å, is shown in Fig. 2 ▸.
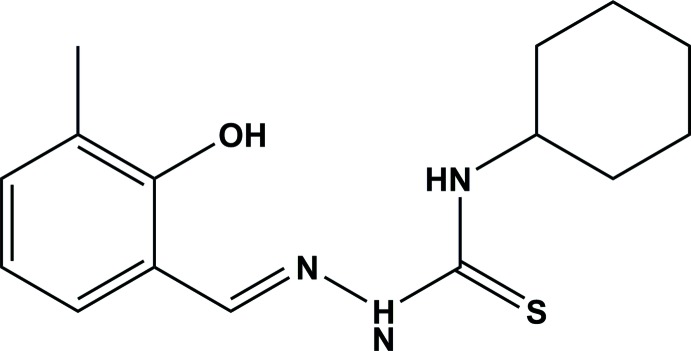


The cyclo­hexane ring (C9–C14) in each mol­ecule has a chair conformation. The mean plane of the four central C atoms (C10/C11/C13/C14) is inclined to the mean plane of the thio­urea moiety [N2—C8(=S1)—N3] by 54.83 (11) and 55.64 (10)° in mol­ecules *A* and *B*, respectively, and by 50.33 (10) and 65.30 (10)° to the benzene rings (C1–C6) in mol­ecules *A* and *B*, respectively. The benzene ring is inclined to the mean plane of the thio­urea moiety by 10.95 (8)° in mol­ecule *A* and 9.80 (8)° in mol­ecule *B*.

**Figure 3:**
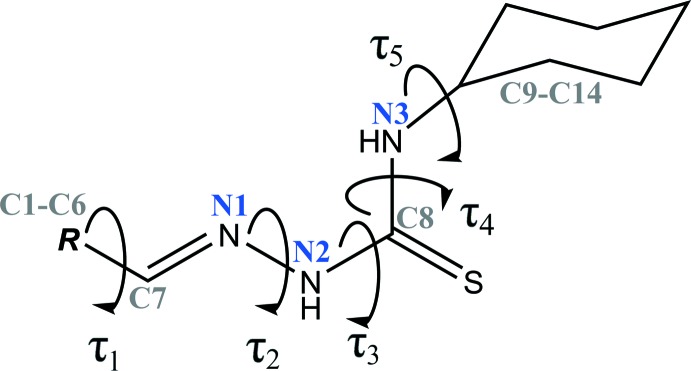
General chemical diagram showing torsion angles, *τ*_1_, *τ*_2_, *τ*_3_, *τ*_4_ and *τ*_5_ in the title compound.

The unique mol­ecular conformations of the two mol­ecules can be characterized by five torsion angles, *i.e. τ*_1_ (C1—C6—C7—N1), *τ*_2_ (C7—N1—N2—C8), *τ*_3_ (N1—N2—C8—N3), *τ*_4_ (N2—C8—N3—C9) and *τ*_5_ (C8—N3—C9—C10), as illustrated in Fig. 3 ▸. The torsion angle *τ*_1_ between the benzyl­idine ring and the azomethine double bond for both mol­ecules are approximately 0° [3.0 (2)° in mol­ecule *A* and 1.9 (2)° in mol­ecule *B*], signifying the coplanarity between benzyl­idine ring and the azomethine double bond (C7=N1). In mol­ecule *B*, the azomethine double bond is close to planar with the hydrazine moiety [*τ*_2_ = 177.23 (14)°], whereas *τ*_2_ in mol­ecule *A* is slightly twisted [*τ*_2_ = 171.68 (14)°]. In both mol­ecules, the torsion angle between the hydrazine moiety and the carbo­thio group are also slight twisted with *τ*_3_ values in mol­ecules *A* and *B* of 7.4 (2) and −10.2 (2)°, respectively. Similarly to *τ*_1_, the carbo­thio group is almost coplanar with the thio­amide group for both mol­ecules, as implied by torsion angle *τ*_4_ [178.07 (14)° in mol­ecule *A* and 175.59 (14)° in mol­ecule *B*], which are approximately 180°. The thio­amide group and the cyclo­hexane ring are almost perpendicular to each other with *τ*_5_ torsion angles of 85.3 (2) and −81.6 (2)° in mol­ecules *A* and *B*, respectively. This may arise from the steric repulsion between the cyclo­hexane ring and adjacent sulfur atom.

## Supra­molecular features   

**Figure 4:**
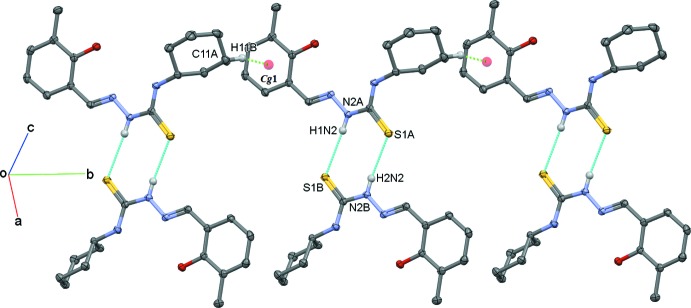
A partial view, normal to the *ac* plane, of the crystal packing of the title compound. The N—H⋯S hydrogen bonds are shown as cyan dotted lines, and the C—H⋯ π inter­actions as green dotted lines (see Table 1 ▸ for details). For clarity, only the hydrogen atoms involved in these inter­actions have been included.

In the crystal, the *A* and *B* mol­ecules are connected into ‘dimers’ with an 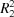
(8) ring motif, *via* N2*A*—H1*N*2⋯S1*B* ^i^ and N2*B*—H2*N*2⋯S1*A* ^i^ hydrogen bonds (Fig. 4 ▸ and Table 1 ▸). The *A* mol­ecules are further linked by a C—H⋯π inter­action, so linking the *A*–*B* units to form ribbons propagating along the *b*-axis direction, as illustrated in Fig. 4 ▸.

## Database survey   

A search of the Cambridge Structural Database (CSD version 5.40, last update February 2019; Groom *et al.*, 2016 ▸) using (*E*)-2-benzyl­idene-*N*-cyclo­hexyl­hydrazine-1-carbo­thio­amide as the reference moiety resulted in nine structures containing a cyclo­hexyl­hydrazinecarbo­thio­amide moiety with different substituents (*R*). The different substituents (*R*) together with the torsion angles of the hydrazinecarbo­thio­amide connecting bridge are compiled in Table 2 ▸ (*cf*. Fig. 3 ▸). In these structures, including the title compound, the hydrazinecarbo­thio­amide connecting bridge is nearly planar as *τ*_2_, *τ*_3_ and *τ*_4_ are in, respectively, anti-periplanar (153.5 to 179.3°), syn-periplanar (0.8 to 14.7°) and anti-periplanar (from 171.8 to 180.0°) conformations. The attached cyclo­hexane ring is always close to perpendicular to the thio­amide group and with a syn/anti-clinal (*τ*_5_ = 78.3 to 94.5°) conformation. Furthermore, torsion angle *τ*_1_ for most of these structures exists in a syn-periplanar conformation, ranging from 0 to 25.8°, but there is one outlier (mol­ecule *B* in NALKOD; Basheer *et al.*, 2016*b* ▸) where torsion angle *τ*_1_ is in a syn-clinal (36.2°) conformation. The cyclo­hexyl­hydrazinecarbo­thio­amide moiety of this structure is substituted with an anthracen-9-yl­methyl­ene ring system.

## Synthesis and crystallization   

**Figure 5:**
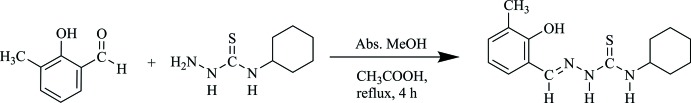
Reaction scheme for the synthesis of the title compound.

The reaction scheme for the synthesis of the title Schiff base compound is given in Fig. 5 ▸.

2-Hy­droxy-3-methyl­benzaldehyde (0.68 g, 5.00 mmol) was dissolved in 20 ml of methanol. Glacial acetic acid (0.20 ml) was added and the mixture was refluxed for 30 min. A solution of *N*-cyclo­hexyl­hydrazine carbo­thio­amide (0.87 g, 5 mmol) in 20 ml methanol was added dropwise with stirring to the aldehyde solution. The resulting colourless solution was refluxed for 4 h with stirring. A colourless precipitate was obtained on evaporation of the solvent. The crude product was washed with *n*-hexane (5 ml). The recovered product was dissolved in aceto­nitrile and purified by recrystallization. Colourless block-like crystals suitable for X-ray diffraction analysis were obtained on slow evaporation of the aceto­nitrile solvent (m.p. 513–514 K, yield 93%).

*Spectroscopic and analytical data*: ^1^H NMR (500 MHz, DMSO-*d*_6_, Me_4_Si ppm): δ 11.27 (*s*, N—NH), δ 9.51 (*s*, OH), δ 8.34 (*s*, HC=N), δ 8.05 (*d*, *J* = 8.35 Hz, CS=NH), δ 7.39–6.81 (multiplet, aromatic-H), δ 2.20 (*s*, Ph—CH_3_), δ 1.87–1.14 (multiplet, cyclo­hexyl-H) ppm. ^13^C NMR (DMSO-*d*_6_, Me_4_Si ppm): δ 175.79 (C=S), δ 154.29 (C=N), δ 143.76-119.17 (C-aromatic), δ 15.93 (CH_3_), δ 52.87–24.90 (C-cyclo­hex­yl) ppm. IR (KBr pellets, cm^−1^): 3364 (NH), 3148 (OH), 2989(CH_3_), 2931 and 2854 (CH, cyclo­hex­yl), 1620 (C=N), 1540 (C=C, aromatic), 1268 (C=S), 1218 (CH, bend., aromatic), 1122 (C—O). 1075 (C—N). Elemental analysis calculated for C_15_H_21_N_3_OS (*M*_r_ = 291.41 g mol^−1^); C, 61.77; H, 7.21; N, 14.42%; found: C, 61.81; H, 7.19; N, 14.42%.

## Refinement   

Crystal data, data collection and structure refinement details are summarized in Table 3 ▸. The O and N-bound H atoms were located in a difference-Fourier map and freely refined. The C-bound H atoms were positioned geometrically and refined using a riding model: C—H = 0.95–1.00 Å with *U*_iso_(H) = 1.5*U*_eq_(C-meth­yl) and 1.2*U*_eq_(C) for other H atoms.

## Supplementary Material

Crystal structure: contains datablock(s) I, Global. DOI: 10.1107/S2056989019008946/su5501sup1.cif

Structure factors: contains datablock(s) I. DOI: 10.1107/S2056989019008946/su5501Isup2.hkl

CCDC reference: 1480651

Additional supporting information:  crystallographic information; 3D view; checkCIF report

## Figures and Tables

**Table 1:** Hydrogen-bond geometry (Å, °) *Cg*1 is the centroid of benzene ring C1*A*–C6*A*.

*D*—H⋯*A*	*D*—H	H⋯*A*	*D*⋯*A*	*D*—H⋯*A*
O1*A*—H1*O*1⋯N1*A*	0.80 (2)	1.98 (2)	2.6844 (19)	146 (2)
O1*B*—H1*O*2⋯N1*B*	0.84 (2)	1.91 (2)	2.664 (2)	148 (2)
N2*A*—H1*N*2⋯S1*B* ^i^	0.85 (2)	2.60 (2)	3.4414 (16)	170 (2)
N2*B*—H2*N*2⋯S1*A* ^i^	0.85 (2)	2.53 (2)	3.3568 (15)	164 (2)
C11*A*—H11*B*⋯*Cg*1^ii^	0.99	2.93	3.801 (2)	148

Symmetry codes: (i) 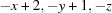; (ii) 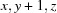.

**Table 2:** Torsion angles τ_1_, τ_2_, τ_3_, τ_4_ and τ_5_ (°)

Compound	*R*	τ_1_	τ_2_	τ_3_	τ_4_	τ_5_
Title compound	2-hy­droxy-3-methyl­benzyl­idene	3.2, 1.9	171.7, 177.2	7.4, 10.2	178.1, 175.6	85.3, 81.6
ABUHEN (Basheer *et al.*, 2017 ▸)	pyren-1-yl­methyl­ene	10.1	174.9	1.2	180.0	81.6
BEFZIY (Basheer *et al.*, 2016*a* ▸)	2-hy­droxy-1-naphth­yl)methyl­ene	0.9	179.3	6.8	176.6	83.4
BEVNAR (Koo *et al.*, 1981 ▸)	4-amino­benzyl­idene	14.3	175.0	7.4	178.5	94.5
LAQCIR (Jacob & Kurup, 2012 ▸)	5-bromo-2-hy­droxy-3-meth­oxy­benzyl­idene	10.1	176.8	4.1	179.5	86.2
LEPFIW (Seena *et al.*, 2006 ▸)	1-(2-hy­droxy­phen­yl)ethyl­idene	3.9, 6.6	155.0, 153.5	14.0, 14.7	175.7, 171.8	91.9, 81.6
NALKOD (Basheer *et al.*, 2016*b* ▸)	anthracen-9-yl­methyl­ene	25.8, 36.2	171.6, 178.6	0.8, 1.4	172.9, 176.2	79.0, 79.2
OBOLOJ (Arafath, 2017*a* ▸)	5-chloro-2-hy­droxy­benzyl­idene	4.7	176.0	5.5	176.7	83.7
XOYKAZ (Bhat *et al.*, 2015 ▸)	4-eth­oxy­benzyl­idene	0.5	169.3	11.6	176.2	85.8
YUXJOS (Arafath *et al.*, 2018 ▸)	3-*t*-butyl-2-hy­droxy­phen­yl)methyl­idene	11.8	170.1	12.5	176.2	78.3

**Note**: The title compound and compounds LEPFIW and NALKOD crystallize with two independent mol­ecules in the asymmetric unit.

**Table 3:** Experimental details

Crystal data	
Chemical formula	C_15_H_21_N_3_OS
*M*_r_	291.41
Crystal system, space group	Triclinic, *P* 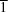
Temperature (K)	100
*a*, *b*, *c* (Å)	10.7799 (11), 10.9481 (11), 14.1895 (15)
α, β, γ (°)	74.526 (2), 68.246 (1), 80.207 (2)
*V* (Å^3^)	1494.2 (3)
*Z*	4
Radiation type	Mo *K*α
μ (mm^−1^)	0.22
Crystal size (mm)	0.34 × 0.14 × 0.10

Data collection	
Diffractometer	Bruker APEXII CCD
Absorption correction	Multi-scan (*SADABS*; Bruker, 2012 ▸)
*T*_min_, *T*_max_	0.873, 0.935
No. of measured, independent and observed [*I* > 2σ(*I*)] reflections	50505, 8135, 5805
*R*_int_	0.069
(sin θ/λ)_max_ (Å^−1^)	0.690

Refinement	
*R*[*F* ^2^ > 2σ(*F* ^2^)], *wR*(*F* ^2^), *S*	0.049, 0.119, 1.04
No. of reflections	8135
No. of parameters	387
H-atom treatment	H atoms treated by a mixture of independent and constrained refinement
Δρ_max_, Δρ_min_ (e Å^−3^)	0.42, −0.36

Computer programs: *APEX2* and *SAINT* (Bruker, 2012 ▸), *SHELXS97* (Sheldrick, 2008 ▸), *SHELXL2013* (Sheldrick, 2015 ▸), *Mercury* (Macrae *et al.*, 2008 ▸) and *PLATON* (Spek, 2009 ▸).

## supplementary crystallographic information

### Crystal data

**Table:**

C_15_H_21_N_3_OS	*Z* = 4
*M**_r_* = 291.41	*F*(000) = 624
Triclinic, *P*1	*D*_x_ = 1.295 Mg m^−^^3^
*a* = 10.7799 (11) Å	Mo *K*α radiation, λ = 0.71073 Å
*b* = 10.9481 (11) Å	Cell parameters from 6929 reflections
*c* = 14.1895 (15) Å	θ = 2.2–29.3°
α = 74.526 (2)°	µ = 0.22 mm^−^^1^
β = 68.246 (1)°	*T* = 100 K
γ = 80.207 (2)°	Block, colourless
*V* = 1494.2 (3) Å^3^	0.34 × 0.14 × 0.10 mm

### Data collection

**Table:**

Bruker APEXII CCD diffractometer	5805 reflections with *I* > 2σ(*I*)
φ and ω scans	*R*_int_ = 0.069
Absorption correction: multi-scan (SADABS; Bruker, 2012)	θ_max_ = 29.4°, θ_min_ = 1.6°
*T*_min_ = 0.873, *T*_max_ = 0.935	*h* = −14→14
50505 measured reflections	*k* = −15→15
8135 independent reflections	*l* = −19→19

### Refinement

**Table:**

Refinement on *F*^2^	Primary atom site location: structure-invariant direct methods
Least-squares matrix: full	Secondary atom site location: difference Fourier map
*R*[*F*^2^ > 2σ(*F*^2^)] = 0.049	Hydrogen site location: mixed
*wR*(*F*^2^) = 0.119	H atoms treated by a mixture of independent and constrained refinement
*S* = 1.03	*w* = 1/[σ^2^(*F*_o_^2^) + (0.0524*P*)^2^ + 0.3685*P*] where *P* = (*F*_o_^2^ + 2*F*_c_^2^)/3
8135 reflections	(Δ/σ)_max_ = 0.001
387 parameters	Δρ_max_ = 0.42 e Å^−^^3^
0 restraints	Δρ_min_ = −0.36 e Å^−^^3^

### Special details

**Table:**

Geometry. All esds (except the esd in the dihedral angle between two l.s. planes) are estimated using the full covariance matrix. The cell esds are taken into account individually in the estimation of esds in distances, angles and torsion angles; correlations between esds in cell parameters are only used when they are defined by crystal symmetry. An approximate (isotropic) treatment of cell esds is used for estimating esds involving l.s. planes.

### Fractional atomic coordinates and isotropic or equivalent isotropic displacement parameters (Å^2^)

**Table:**

	*x*	*y*	*z*	*U*_iso_*/*U*_eq_	
S1A	0.69733 (4)	0.60902 (4)	0.10987 (3)	0.01869 (11)	
O1A	0.20574 (12)	0.29193 (12)	0.27567 (9)	0.0204 (3)	
H1O1	0.264 (2)	0.340 (2)	0.2474 (19)	0.047 (7)*	
N1A	0.44459 (13)	0.36989 (12)	0.14267 (10)	0.0157 (3)	
N2A	0.54998 (14)	0.44583 (13)	0.10122 (11)	0.0168 (3)	
H1N2	0.617 (2)	0.4267 (19)	0.0507 (16)	0.032 (6)*	
N3A	0.45512 (13)	0.54155 (13)	0.23929 (11)	0.0180 (3)	
H1N3	0.387 (2)	0.5075 (19)	0.2500 (16)	0.031 (6)*	
C1A	0.23068 (15)	0.20058 (15)	0.22090 (12)	0.0166 (3)	
C2A	0.13421 (16)	0.11183 (15)	0.25585 (13)	0.0180 (3)	
C3A	0.15680 (17)	0.01785 (16)	0.20070 (13)	0.0212 (4)	
H3AA	0.0925	−0.0427	0.2231	0.025*	
C4A	0.27061 (17)	0.00986 (16)	0.11389 (14)	0.0219 (4)	
H4AA	0.2840	−0.0557	0.0779	0.026*	
C5A	0.36387 (17)	0.09792 (16)	0.08048 (13)	0.0204 (3)	
H5AA	0.4412	0.0933	0.0206	0.024*	
C6A	0.34648 (16)	0.19418 (15)	0.13340 (12)	0.0163 (3)	
C7A	0.45032 (16)	0.28267 (15)	0.09523 (13)	0.0173 (3)	
H7AA	0.5243	0.2759	0.0337	0.021*	
C8A	0.55885 (15)	0.52858 (15)	0.15423 (12)	0.0154 (3)	
C9A	0.44553 (15)	0.62215 (16)	0.30920 (12)	0.0175 (3)	
H9AA	0.5368	0.6226	0.3119	0.021*	
C10A	0.39567 (17)	0.75839 (16)	0.27188 (14)	0.0223 (4)	
H10A	0.3072	0.7594	0.2653	0.027*	
H10B	0.4588	0.7950	0.2024	0.027*	
C11A	0.38338 (17)	0.83900 (17)	0.34832 (14)	0.0249 (4)	
H11A	0.4734	0.8448	0.3495	0.030*	
H11B	0.3465	0.9261	0.3246	0.030*	
C12A	0.29295 (17)	0.78223 (19)	0.45741 (15)	0.0300 (4)	
H12A	0.2924	0.8328	0.5059	0.036*	
H12B	0.2002	0.7864	0.4580	0.036*	
C13A	0.3395 (2)	0.64536 (19)	0.49414 (14)	0.0334 (5)	
H13A	0.2742	0.6092	0.5627	0.040*	
H13B	0.4269	0.6425	0.5031	0.040*	
C14A	0.35411 (18)	0.56458 (17)	0.41752 (13)	0.0253 (4)	
H14A	0.3914	0.4777	0.4415	0.030*	
H14B	0.2648	0.5581	0.4153	0.030*	
C15A	0.01137 (16)	0.12174 (18)	0.34918 (14)	0.0246 (4)	
H15A	−0.0495	0.0589	0.3588	0.037*	
H15B	−0.0337	0.2073	0.3387	0.037*	
H15C	0.0371	0.1055	0.4111	0.037*	
S1B	1.20980 (4)	0.63000 (4)	0.11943 (3)	0.01957 (11)	
O1B	0.72600 (12)	0.31105 (11)	0.27336 (9)	0.0204 (3)	
H1O2	0.790 (2)	0.358 (2)	0.2455 (18)	0.042 (7)*	
N1B	0.96393 (13)	0.38885 (13)	0.14385 (10)	0.0165 (3)	
N2B	1.07086 (14)	0.46229 (13)	0.10739 (11)	0.0183 (3)	
H2N2	1.141 (2)	0.4453 (19)	0.0585 (17)	0.035 (6)*	
N3B	0.95163 (13)	0.58802 (14)	0.22201 (11)	0.0191 (3)	
H2N3	0.8858 (19)	0.5517 (18)	0.2277 (14)	0.023 (5)*	
C1B	0.74994 (15)	0.22243 (15)	0.21634 (12)	0.0160 (3)	
C2B	0.64909 (16)	0.14034 (16)	0.24528 (13)	0.0182 (3)	
C3B	0.67030 (16)	0.04933 (16)	0.18792 (13)	0.0212 (4)	
H3BA	0.6019	−0.0055	0.2052	0.025*	
C4B	0.78870 (17)	0.03605 (16)	0.10606 (14)	0.0221 (4)	
H4BA	0.8018	−0.0285	0.0692	0.027*	
C5B	0.88741 (16)	0.11750 (16)	0.07866 (13)	0.0198 (3)	
H5BA	0.9683	0.1087	0.0224	0.024*	
C6B	0.87008 (15)	0.21254 (15)	0.13242 (12)	0.0168 (3)	
C7B	0.97581 (16)	0.29730 (15)	0.09955 (13)	0.0178 (3)	
H7BA	1.0559	0.2846	0.0438	0.021*	
C8B	1.06817 (16)	0.55741 (15)	0.15303 (12)	0.0165 (3)	
C9B	0.93058 (15)	0.68143 (15)	0.28450 (12)	0.0171 (3)	
H9BA	0.9867	0.7537	0.2406	0.021*	
C10B	0.97252 (16)	0.62377 (16)	0.37971 (13)	0.0200 (3)	
H10C	1.0684	0.5927	0.3570	0.024*	
H10D	0.9197	0.5504	0.4230	0.024*	
C11B	0.95005 (16)	0.72239 (16)	0.44436 (13)	0.0214 (4)	
H11C	0.9730	0.6819	0.5077	0.026*	
H11D	1.0100	0.7914	0.4034	0.026*	
C12B	0.80487 (16)	0.77855 (17)	0.47574 (13)	0.0219 (4)	
H12C	0.7460	0.7118	0.5248	0.026*	
H12D	0.7953	0.8471	0.5120	0.026*	
C13B	0.76067 (17)	0.83197 (17)	0.38142 (14)	0.0224 (4)	
H13C	0.8111	0.9065	0.3372	0.027*	
H13D	0.6642	0.8610	0.4051	0.027*	
C14B	0.78417 (15)	0.73274 (16)	0.31723 (13)	0.0201 (4)	
H14C	0.7268	0.6620	0.3590	0.024*	
H14D	0.7592	0.7717	0.2546	0.024*	
C15B	0.52399 (16)	0.15270 (17)	0.33654 (14)	0.0237 (4)	
H15D	0.4569	0.1011	0.3383	0.036*	
H15E	0.4886	0.2420	0.3299	0.036*	
H15F	0.5447	0.1231	0.4010	0.036*	

### Atomic displacement parameters (Å^2^)

**Table:**

	*U*^11^	*U*^22^	*U*^33^	*U*^12^	*U*^13^	*U*^23^
S1A	0.01576 (19)	0.0224 (2)	0.0179 (2)	−0.00439 (16)	−0.00298 (16)	−0.00655 (17)
O1A	0.0200 (6)	0.0223 (7)	0.0185 (6)	−0.0050 (5)	−0.0019 (5)	−0.0086 (5)
N1A	0.0150 (6)	0.0159 (7)	0.0166 (7)	−0.0018 (5)	−0.0052 (5)	−0.0041 (5)
N2A	0.0155 (6)	0.0188 (7)	0.0155 (7)	−0.0032 (5)	−0.0014 (6)	−0.0072 (6)
N3A	0.0142 (6)	0.0223 (8)	0.0187 (7)	−0.0050 (6)	−0.0020 (6)	−0.0095 (6)
C1A	0.0199 (8)	0.0162 (8)	0.0153 (8)	−0.0006 (6)	−0.0079 (6)	−0.0036 (6)
C2A	0.0177 (7)	0.0191 (8)	0.0173 (8)	−0.0022 (6)	−0.0080 (6)	−0.0012 (7)
C3A	0.0238 (8)	0.0177 (9)	0.0244 (9)	−0.0054 (7)	−0.0118 (7)	−0.0013 (7)
C4A	0.0291 (9)	0.0168 (9)	0.0238 (9)	−0.0012 (7)	−0.0122 (7)	−0.0070 (7)
C5A	0.0238 (8)	0.0177 (9)	0.0187 (9)	0.0002 (7)	−0.0056 (7)	−0.0061 (7)
C6A	0.0197 (8)	0.0146 (8)	0.0151 (8)	−0.0009 (6)	−0.0074 (6)	−0.0025 (6)
C7A	0.0180 (7)	0.0175 (8)	0.0146 (8)	−0.0015 (6)	−0.0032 (6)	−0.0043 (6)
C8A	0.0158 (7)	0.0158 (8)	0.0153 (8)	−0.0003 (6)	−0.0072 (6)	−0.0024 (6)
C9A	0.0162 (7)	0.0223 (9)	0.0159 (8)	−0.0045 (6)	−0.0036 (6)	−0.0081 (7)
C10A	0.0248 (8)	0.0215 (9)	0.0225 (9)	−0.0012 (7)	−0.0091 (7)	−0.0072 (7)
C11A	0.0223 (8)	0.0248 (10)	0.0327 (10)	0.0003 (7)	−0.0109 (8)	−0.0142 (8)
C12A	0.0206 (8)	0.0435 (12)	0.0320 (11)	−0.0055 (8)	−0.0022 (8)	−0.0262 (9)
C13A	0.0415 (11)	0.0405 (12)	0.0187 (10)	−0.0133 (9)	−0.0027 (8)	−0.0120 (8)
C14A	0.0295 (9)	0.0272 (10)	0.0180 (9)	−0.0086 (8)	−0.0031 (7)	−0.0066 (7)
C15A	0.0198 (8)	0.0282 (10)	0.0244 (9)	−0.0067 (7)	−0.0040 (7)	−0.0056 (8)
S1B	0.01561 (19)	0.0223 (2)	0.0206 (2)	−0.00340 (16)	−0.00332 (16)	−0.00756 (17)
O1B	0.0204 (6)	0.0208 (6)	0.0198 (6)	−0.0032 (5)	−0.0019 (5)	−0.0106 (5)
N1B	0.0154 (6)	0.0169 (7)	0.0173 (7)	−0.0020 (5)	−0.0052 (5)	−0.0042 (6)
N2B	0.0155 (7)	0.0203 (8)	0.0177 (7)	−0.0029 (6)	−0.0013 (6)	−0.0074 (6)
N3B	0.0148 (6)	0.0217 (8)	0.0227 (8)	−0.0024 (6)	−0.0035 (6)	−0.0117 (6)
C1B	0.0185 (7)	0.0145 (8)	0.0151 (8)	0.0014 (6)	−0.0067 (6)	−0.0039 (6)
C2B	0.0179 (7)	0.0185 (8)	0.0176 (8)	−0.0002 (6)	−0.0072 (6)	−0.0022 (7)
C3B	0.0217 (8)	0.0200 (9)	0.0244 (9)	−0.0034 (7)	−0.0103 (7)	−0.0047 (7)
C4B	0.0275 (9)	0.0198 (9)	0.0246 (9)	−0.0003 (7)	−0.0117 (7)	−0.0109 (7)
C5B	0.0208 (8)	0.0211 (9)	0.0176 (8)	0.0000 (7)	−0.0041 (7)	−0.0095 (7)
C6B	0.0179 (7)	0.0171 (8)	0.0163 (8)	−0.0011 (6)	−0.0066 (6)	−0.0042 (6)
C7B	0.0175 (7)	0.0186 (8)	0.0158 (8)	0.0003 (6)	−0.0042 (6)	−0.0049 (7)
C8B	0.0186 (7)	0.0160 (8)	0.0142 (8)	−0.0011 (6)	−0.0059 (6)	−0.0021 (6)
C9B	0.0178 (7)	0.0179 (8)	0.0167 (8)	−0.0032 (6)	−0.0035 (6)	−0.0079 (7)
C10B	0.0183 (8)	0.0215 (9)	0.0199 (9)	0.0017 (6)	−0.0066 (7)	−0.0063 (7)
C11B	0.0228 (8)	0.0252 (9)	0.0188 (9)	−0.0011 (7)	−0.0096 (7)	−0.0063 (7)
C12B	0.0225 (8)	0.0247 (9)	0.0196 (9)	−0.0005 (7)	−0.0062 (7)	−0.0092 (7)
C13B	0.0207 (8)	0.0245 (9)	0.0262 (10)	0.0035 (7)	−0.0107 (7)	−0.0121 (8)
C14B	0.0179 (8)	0.0236 (9)	0.0218 (9)	−0.0006 (7)	−0.0081 (7)	−0.0087 (7)
C15B	0.0199 (8)	0.0253 (9)	0.0231 (9)	−0.0036 (7)	−0.0036 (7)	−0.0048 (7)

### Geometric parameters (Å, º)

**Table:**

S1A—C8A	1.6897 (15)	S1B—C8B	1.6914 (16)
O1A—C1A	1.3583 (19)	O1B—C1B	1.3569 (19)
O1A—H1O1	0.80 (2)	O1B—H1O2	0.83 (2)
N1A—C7A	1.289 (2)	N1B—C7B	1.284 (2)
N1A—N2A	1.3758 (18)	N1B—N2B	1.3762 (18)
N2A—C8A	1.357 (2)	N2B—C8B	1.357 (2)
N2A—H1N2	0.85 (2)	N2B—H2N2	0.85 (2)
N3A—C8A	1.328 (2)	N3B—C8B	1.330 (2)
N3A—C9A	1.461 (2)	N3B—C9B	1.463 (2)
N3A—H1N3	0.82 (2)	N3B—H2N3	0.840 (19)
C1A—C6A	1.404 (2)	C1B—C2B	1.401 (2)
C1A—C2A	1.406 (2)	C1B—C6B	1.409 (2)
C2A—C3A	1.390 (2)	C2B—C3B	1.387 (2)
C2A—C15A	1.499 (2)	C2B—C15B	1.500 (2)
C3A—C4A	1.390 (2)	C3B—C4B	1.388 (2)
C3A—H3AA	0.9500	C3B—H3BA	0.9500
C4A—C5A	1.378 (2)	C4B—C5B	1.382 (2)
C4A—H4AA	0.9500	C4B—H4BA	0.9500
C5A—C6A	1.400 (2)	C5B—C6B	1.397 (2)
C5A—H5AA	0.9500	C5B—H5BA	0.9500
C6A—C7A	1.458 (2)	C6B—C7B	1.453 (2)
C7A—H7AA	0.9500	C7B—H7BA	0.9500
C9A—C14A	1.517 (2)	C9B—C14B	1.522 (2)
C9A—C10A	1.520 (2)	C9B—C10B	1.526 (2)
C9A—H9AA	1.0000	C9B—H9BA	1.0000
C10A—C11A	1.529 (2)	C10B—C11B	1.530 (2)
C10A—H10A	0.9900	C10B—H10C	0.9900
C10A—H10B	0.9900	C10B—H10D	0.9900
C11A—C12A	1.519 (3)	C11B—C12B	1.526 (2)
C11A—H11A	0.9900	C11B—H11C	0.9900
C11A—H11B	0.9900	C11B—H11D	0.9900
C12A—C13A	1.513 (3)	C12B—C13B	1.523 (2)
C12A—H12A	0.9900	C12B—H12C	0.9900
C12A—H12B	0.9900	C12B—H12D	0.9900
C13A—C14A	1.526 (2)	C13B—C14B	1.529 (2)
C13A—H13A	0.9900	C13B—H13C	0.9900
C13A—H13B	0.9900	C13B—H13D	0.9900
C14A—H14A	0.9900	C14B—H14C	0.9900
C14A—H14B	0.9900	C14B—H14D	0.9900
C15A—H15A	0.9800	C15B—H15D	0.9800
C15A—H15B	0.9800	C15B—H15E	0.9800
C15A—H15C	0.9800	C15B—H15F	0.9800
			
C1A—O1A—H1O1	108.6 (17)	C1B—O1B—H1O2	107.4 (15)
C7A—N1A—N2A	116.82 (13)	C7B—N1B—N2B	116.97 (14)
C8A—N2A—N1A	119.82 (13)	C8B—N2B—N1B	120.46 (14)
C8A—N2A—H1N2	120.7 (13)	C8B—N2B—H2N2	119.4 (14)
N1A—N2A—H1N2	117.8 (14)	N1B—N2B—H2N2	120.0 (14)
C8A—N3A—C9A	125.71 (13)	C8B—N3B—C9B	124.99 (13)
C8A—N3A—H1N3	117.1 (14)	C8B—N3B—H2N3	116.0 (13)
C9A—N3A—H1N3	116.9 (14)	C9B—N3B—H2N3	118.9 (13)
O1A—C1A—C6A	122.24 (14)	O1B—C1B—C2B	116.63 (14)
O1A—C1A—C2A	116.68 (14)	O1B—C1B—C6B	122.02 (14)
C6A—C1A—C2A	121.08 (15)	C2B—C1B—C6B	121.35 (15)
C3A—C2A—C1A	117.85 (15)	C3B—C2B—C1B	117.95 (15)
C3A—C2A—C15A	122.40 (14)	C3B—C2B—C15B	122.57 (15)
C1A—C2A—C15A	119.74 (15)	C1B—C2B—C15B	119.48 (15)
C4A—C3A—C2A	122.00 (15)	C2B—C3B—C4B	121.87 (15)
C4A—C3A—H3AA	119.0	C2B—C3B—H3BA	119.1
C2A—C3A—H3AA	119.0	C4B—C3B—H3BA	119.1
C5A—C4A—C3A	119.37 (16)	C5B—C4B—C3B	119.48 (15)
C5A—C4A—H4AA	120.3	C5B—C4B—H4BA	120.3
C3A—C4A—H4AA	120.3	C3B—C4B—H4BA	120.3
C4A—C5A—C6A	121.01 (16)	C4B—C5B—C6B	120.98 (16)
C4A—C5A—H5AA	119.5	C4B—C5B—H5BA	119.5
C6A—C5A—H5AA	119.5	C6B—C5B—H5BA	119.5
C5A—C6A—C1A	118.68 (14)	C5B—C6B—C1B	118.35 (14)
C5A—C6A—C7A	118.22 (15)	C5B—C6B—C7B	118.96 (15)
C1A—C6A—C7A	123.09 (14)	C1B—C6B—C7B	122.70 (14)
N1A—C7A—C6A	121.83 (15)	N1B—C7B—C6B	121.80 (15)
N1A—C7A—H7AA	119.1	N1B—C7B—H7BA	119.1
C6A—C7A—H7AA	119.1	C6B—C7B—H7BA	119.1
N3A—C8A—N2A	116.73 (14)	N3B—C8B—N2B	116.78 (14)
N3A—C8A—S1A	123.76 (12)	N3B—C8B—S1B	124.07 (12)
N2A—C8A—S1A	119.51 (12)	N2B—C8B—S1B	119.15 (12)
N3A—C9A—C14A	108.61 (13)	N3B—C9B—C14B	109.69 (12)
N3A—C9A—C10A	112.01 (13)	N3B—C9B—C10B	111.20 (13)
C14A—C9A—C10A	111.06 (14)	C14B—C9B—C10B	110.58 (13)
N3A—C9A—H9AA	108.4	N3B—C9B—H9BA	108.4
C14A—C9A—H9AA	108.4	C14B—C9B—H9BA	108.4
C10A—C9A—H9AA	108.4	C10B—C9B—H9BA	108.4
C9A—C10A—C11A	110.55 (14)	C9B—C10B—C11B	110.68 (13)
C9A—C10A—H10A	109.5	C9B—C10B—H10C	109.5
C11A—C10A—H10A	109.5	C11B—C10B—H10C	109.5
C9A—C10A—H10B	109.5	C9B—C10B—H10D	109.5
C11A—C10A—H10B	109.5	C11B—C10B—H10D	109.5
H10A—C10A—H10B	108.1	H10C—C10B—H10D	108.1
C12A—C11A—C10A	111.31 (14)	C12B—C11B—C10B	111.23 (13)
C12A—C11A—H11A	109.4	C12B—C11B—H11C	109.4
C10A—C11A—H11A	109.4	C10B—C11B—H11C	109.4
C12A—C11A—H11B	109.4	C12B—C11B—H11D	109.4
C10A—C11A—H11B	109.4	C10B—C11B—H11D	109.4
H11A—C11A—H11B	108.0	H11C—C11B—H11D	108.0
C13A—C12A—C11A	111.40 (15)	C13B—C12B—C11B	111.49 (14)
C13A—C12A—H12A	109.3	C13B—C12B—H12C	109.3
C11A—C12A—H12A	109.3	C11B—C12B—H12C	109.3
C13A—C12A—H12B	109.3	C13B—C12B—H12D	109.3
C11A—C12A—H12B	109.3	C11B—C12B—H12D	109.3
H12A—C12A—H12B	108.0	H12C—C12B—H12D	108.0
C12A—C13A—C14A	111.91 (16)	C12B—C13B—C14B	111.59 (14)
C12A—C13A—H13A	109.2	C12B—C13B—H13C	109.3
C14A—C13A—H13A	109.2	C14B—C13B—H13C	109.3
C12A—C13A—H13B	109.2	C12B—C13B—H13D	109.3
C14A—C13A—H13B	109.2	C14B—C13B—H13D	109.3
H13A—C13A—H13B	107.9	H13C—C13B—H13D	108.0
C9A—C14A—C13A	111.02 (14)	C9B—C14B—C13B	110.42 (13)
C9A—C14A—H14A	109.4	C9B—C14B—H14C	109.6
C13A—C14A—H14A	109.4	C13B—C14B—H14C	109.6
C9A—C14A—H14B	109.4	C9B—C14B—H14D	109.6
C13A—C14A—H14B	109.4	C13B—C14B—H14D	109.6
H14A—C14A—H14B	108.0	H14C—C14B—H14D	108.1
C2A—C15A—H15A	109.5	C2B—C15B—H15D	109.5
C2A—C15A—H15B	109.5	C2B—C15B—H15E	109.5
H15A—C15A—H15B	109.5	H15D—C15B—H15E	109.5
C2A—C15A—H15C	109.5	C2B—C15B—H15F	109.5
H15A—C15A—H15C	109.5	H15D—C15B—H15F	109.5
H15B—C15A—H15C	109.5	H15E—C15B—H15F	109.5
			
C7A—N1A—N2A—C8A	−171.68 (14)	C7B—N1B—N2B—C8B	−177.23 (14)
O1A—C1A—C2A—C3A	179.53 (14)	O1B—C1B—C2B—C3B	179.53 (14)
C6A—C1A—C2A—C3A	−0.3 (2)	C6B—C1B—C2B—C3B	−0.7 (2)
O1A—C1A—C2A—C15A	0.2 (2)	O1B—C1B—C2B—C15B	−1.0 (2)
C6A—C1A—C2A—C15A	−179.68 (15)	C6B—C1B—C2B—C15B	178.77 (15)
C1A—C2A—C3A—C4A	0.3 (2)	C1B—C2B—C3B—C4B	1.9 (2)
C15A—C2A—C3A—C4A	179.61 (16)	C15B—C2B—C3B—C4B	−177.57 (16)
C2A—C3A—C4A—C5A	−0.5 (3)	C2B—C3B—C4B—C5B	−1.7 (3)
C3A—C4A—C5A—C6A	0.8 (3)	C3B—C4B—C5B—C6B	0.3 (3)
C4A—C5A—C6A—C1A	−0.9 (2)	C4B—C5B—C6B—C1B	0.8 (2)
C4A—C5A—C6A—C7A	178.73 (15)	C4B—C5B—C6B—C7B	−178.94 (15)
O1A—C1A—C6A—C5A	−179.23 (14)	O1B—C1B—C6B—C5B	179.16 (14)
C2A—C1A—C6A—C5A	0.6 (2)	C2B—C1B—C6B—C5B	−0.6 (2)
O1A—C1A—C6A—C7A	1.2 (2)	O1B—C1B—C6B—C7B	−1.1 (2)
C2A—C1A—C6A—C7A	−178.96 (14)	C2B—C1B—C6B—C7B	179.12 (15)
N2A—N1A—C7A—C6A	178.28 (13)	N2B—N1B—C7B—C6B	179.20 (14)
C5A—C6A—C7A—N1A	−176.61 (15)	C5B—C6B—C7B—N1B	177.83 (15)
C1A—C6A—C7A—N1A	3.0 (2)	C1B—C6B—C7B—N1B	−1.9 (2)
C9A—N3A—C8A—N2A	178.07 (14)	C9B—N3B—C8B—N2B	175.59 (14)
C9A—N3A—C8A—S1A	−2.3 (2)	C9B—N3B—C8B—S1B	−4.8 (2)
N1A—N2A—C8A—N3A	−7.4 (2)	N1B—N2B—C8B—N3B	−10.2 (2)
N1A—N2A—C8A—S1A	172.92 (11)	N1B—N2B—C8B—S1B	170.21 (11)
C8A—N3A—C9A—C14A	−151.70 (16)	C8B—N3B—C9B—C14B	155.77 (15)
C8A—N3A—C9A—C10A	85.26 (19)	C8B—N3B—C9B—C10B	−81.60 (19)
N3A—C9A—C10A—C11A	178.51 (13)	N3B—C9B—C10B—C11B	179.99 (13)
C14A—C9A—C10A—C11A	56.87 (18)	C14B—C9B—C10B—C11B	−57.90 (17)
C9A—C10A—C11A—C12A	−56.18 (19)	C9B—C10B—C11B—C12B	55.88 (18)
C10A—C11A—C12A—C13A	54.83 (19)	C10B—C11B—C12B—C13B	−54.08 (19)
C11A—C12A—C13A—C14A	−54.1 (2)	C11B—C12B—C13B—C14B	54.32 (19)
N3A—C9A—C14A—C13A	−179.67 (15)	N3B—C9B—C14B—C13B	−179.21 (14)
C10A—C9A—C14A—C13A	−56.06 (19)	C10B—C9B—C14B—C13B	57.79 (18)
C12A—C13A—C14A—C9A	54.7 (2)	C12B—C13B—C14B—C9B	−56.10 (19)

### Hydrogen-bond geometry (Å, º)

*Cg*1 is the centroid of benzene ring C1*A*–C6*A*.

**Table:**

*D*—H···*A*	*D*—H	H···*A*	*D*···*A*	*D*—H···*A*
O1*A*—H1*O*1···N1*A*	0.80 (2)	1.98 (2)	2.6844 (19)	146 (2)
O1*B*—H1*O*2···N1*B*	0.84 (2)	1.91 (2)	2.664 (2)	148 (2)
N2*A*—H1*N*2···S1*B*^i^	0.85 (2)	2.60 (2)	3.4414 (16)	170 (2)
N2*B*—H2*N*2···S1*A*^i^	0.85 (2)	2.53 (2)	3.3568 (15)	164 (2)
C11*A*—H11*B*···*Cg*1^ii^	0.99	2.93	3.801 (2)	148

Symmetry codes: (i) −*x*+2, −*y*+1, −*z*; (ii) *x*, *y*+1, *z*.
